# Social acceptability of treatments for adolescent idiopathic scoliosis: a cross-sectional study

**DOI:** 10.1186/1748-7161-1-14

**Published:** 2006-08-24

**Authors:** Stefano Negrini, Roberta Carabalona

**Affiliations:** 1 ISICO (Italian Scientific Spine Institute), Via Carlo Crivelli 20, 20122 Milan, Italy; 2 Don Carlo Gnocchi Foundation ONLUS, Care & Research Institute, Via Capecelatro 66, 20148 Milan, Italy

## Abstract

**Background:**

There are no data on social acceptability of scoliosis. Aim. To elicit evidence-based opinions on therapeutic strategies for adolescent idiopathic scoliosis in a sample of families with not affected children, so to understand the social perception of this issue.

**Methods:**

Design. Cross-sectional study. Setting. Secondary schools in 4 northern Italian regions. Participants. Parents of children in the age group at risk of and not affected by scoliosis (Pre-test group = 100, Study group = 3,162). Interventions. Questionnaire: five specific and evidence-based questions regarding scoliosis treatment options and a socio-demographic section. Methodology. "Role-playing" in which it was required to normal people to answer what they would have chosen if they had been in the situation proposed. Main outcome measures. Perception of acceptability of treatments for adolescent idiopathic scoliosis in the general population (social acceptability)

**Results:**

The families support the use of screening (94.8%) at school, immediate bracing (76.4%) for scoliosis with a 60% risk of progression, but also therapeutic exercises (86.9%) in cases with a 25% risk of progression.

**Conclusion:**

There is a growing tendency to consider not only the efficacy, effectiveness and efficiency of treatments, but also their acceptability. This patient-centred aspect is especially more important in areas (like adolescent idiopathic scoliosis) in which there is some evidence on the efficacy of treatments, but not strong and definitive (RCTs). Adolescent idiopathic scoliosis treatments should thus be carefully considered also in the light of their social acceptability.

## Background

Our everyday practice seems to suggest that people prefer, for their children with scoliosis, prevention instead of a "wait and see" approach, even if this prevention of progression implies efforts, expenses, time, and obviously a possible failure. We planned a study to verify this hypothesis.

Children are our future, and therapeutic decisions should reflect this fact: children are not young adults and prevention should be a very important aspect of their care [1].

The long-term natural history of adolescent idiopathic scoliosis (AIS) has recently been described [2,3] : patients with severe curves have a greater incidence of back pain than normal subjects, and reveal minor disabilities, with some degree of deformity and cosmetic concerns. There currently exists some evidence, but not strong and definitive (RCTs), demonstrating the efficacy either of conservative or of surgical AIS treatments [4-6]: when compared to a matched normal sample 20 years after treatment, both braced and surgically treated AIS patients had the same function [7,8], quality of life [9], marital status and number of children [10] as the controls, but experienced more back pain [7,8], a progression of pathology [7,8], and limitations of social activities [9] and sexual function [10] (both categories of limitation being more marked in the surgically-treated than in the braced group [9,10]). Bracing, whose efficacy has been shown [11-13], causes transient disability and has a psychological impact [4,8,9,14-16]; surgery halts progression, but fuses the spine, eliminating its function, and can give rise to complications [7,9,17]. There are not conclusive evidences on therapeutic exercises for AIS [18,11,13,19], and school screening has been widely criticized [15,20] even if can be supported [11,21,22].

This lack of strong (RCT's) evidence on natural history and long-term treatment results has prompted a new approach to the making of treatment decisions, with efficacy, effectiveness and efficiency being accompanied by other elements in which patient preferences play a crucial role. It is important both to be aware that treatment decision-making dynamics can alter in the course of the physician-patient relationship, and also to question of the paternalistic model in which the physician plays the dominant role [23]. Several models of physician-patient interaction are discussed in the literature[23] as well as the complexity of patient participation in decision making [24]. As pointed out by Ford et al.[24] "the concept of 'patient choice' originates from the doctrine of informed consent" but efforts are now geared at combining evidence-based medicine (EBM) with patient-centred care to create an evidence-based patient choice (EBPC) approach [25].

Empirical research in this field is mostly devoted to extreme scenarios (in the case of AIS, to decisions regarding surgery [17]) but the problem of eliciting patient preferences is a focus of growing attention. Acceptability can be viewed from a personal perspective, considering the single patient, but also from a wider perspective, i.e., that of the general population (social acceptability). This concept of social acceptability has been applied mainly to the relationship between society and disabled people[26], but it is also applicable to treatment options [25].

The aim of this study was therefore to elicit treatment preferences from the parents of children in the age group at risk of AIS, in order to consider the question of social acceptability: what do families think about the different therapeutic and preventive options for AIS, once they have been made aware of available evidence, advantages and disadvantages of each treatment?

## Methods

We considered children in the age group at risk of AIS, choosing a population of 11–14 year old secondary school pupils. We selected only those not affected by AIS, in order to avoid biased responses (external influences, decisions already reached, etc.) and/or interference with clinical treatments in progress, and administered a questionnaire to their parents. The Study Group (SG) included the 3,162 families of all the children attending a convenience sample of 10 secondary schools in 4 northern Italian regions (Lombardy, Liguria, Veneto and Piedmont).

We chose the instrument of a questionnaire to reach an ample general sample and to avoid as much as possible a direct interference with the population as it could have been happened through educational expert sessions: the questionnaire had to contain all decisional tools. We created a new questionnaire, which included a socio-demographic section and five specific questions (Tables 1 – Appendixes 1–2). The decision making tools have been chosen on the basis of current knowledge and consensus in this field [4-6,13,19,20,27,28] so to simulate a situation of EBPC in a real role-playing.

**Table 1:** Structure of the questionnaire. AIS: adolescent idiopathic scoliosis. In the rows have been listed: situation (the hypothetical situation in which the responders had to imagine to be before giving their answer), alternatives (hypothetical possibilities in the given situation), decision-making tools (information given to responders to be able to answer), question – which strategy ? (possible choices for the answer).

**Situation**	**Alternatives**	**Decision-making tools**	**Question Which strategy?**
AIS with a 25% risk of progression; consequence if it progresses: bracing until growth is complete	• Do nothing, periodic check-ups Specific physical exercises until growth is complete, in an attempt to prevent progression	• scientific evidence (no proof for or against physical exercises as means of preventing progression) • advantages and disadvantages of each alternative	• Physical exercises until growth is complete, in an attempt to prevent/delay bracing • Periodic check-ups, bracing only if scoliosis progresses
AIS with a 60% risk of progression; consequence if it progresses: surgical correction	• Do nothing, periodic check-ups • Bracing until growth is complete	• scientific evidence (proof of the efficacy of bracing, but not in all cases) • advantages and disadvantages of each alternative	• Immediate bracing, in order to avoid surgical correction • Periodic check-ups, bracing only if scoliosis progresses • No bracing at all, periodic check-ups, surgical correction if scoliosis progresses
School screening for AIS	• not to screen • to screen	• Scientific evidence (absence of consensus): pros and cons	• not to screen • to screen

With the aim of validating the questionnaire, we performed both a pre-test survey involving 100 parents (Pre-test Group: PG) (Table 2) recruited during a school meeting and a test-retest on 18 subjects in order to evaluate the comprehensibility and repeatability of our questionnaire. At the end of the pre-test survey, we added a single question "What is your opinion on the questionnaire ?" that had a four-point ordinal scale answer: "1. easy to understand and answer; 2. complex to understand and easy to answer; 3. complex to understand and difficult to answer; 4. incomprehensible".

**Table 2:** Population characteristics and results in the full and pre-test groups: percentages (95% confidence interval) are reported. AIS: adolescent idiopathic scoliosis.

		**Study Sroup (SG)**	**Pre-test Group (PG)**
Sample		3162	100
Gender	Females	76.15%	83%
	Males	13.85%	17%
Age	Average	41.2	37.7
	Range	28–62	29–49
Education	primary	4.37%	5%
	secondary	32.71%	28%
	high-school	51.28%	58%
	university	11.64%	9%

While in the PG the pre-test methodology required a direct interaction with families sample with the only aim of verifying if they understood completely the questionnaire (sometime giving more details and clarifications) and not adding any more information, in the SG the questionnaire was given by the teachers at school together with and headmaster's letter asking children to answer at home with their parents, with restitution the following day (Table 2).

The questionnaires have been collected between March and April 2002.

### Statistical analysis

Repeatability was assessed with percent agreement, whereas PG and SG data were analysed using percentages (pointwise and 95% confidence interval estimation). Statistical association has been assessed using χ^2 ^test (level of significance 0.05). Software used: Excel 7.0.

## Results

The questionnaire was considered easy by 8%, complex by 84%, complex and difficult by 8%, and incomprehensible by none of the responders. Test-retest analysis showed agreement ranging from 68.8% to 88.9%.

The overall SG response rate was 34% (1,075 responders) and the inter-school response rate ranged from 10 to 67.5%: an increasing no-response trend (from 1.3% to 3.4%) from the first to the last question was observed. Due to the low response rate obtained, we compared the results of the SG to those of the PG, in which a 100% response rate had been obtained (Table 2).

The families were found to support the use of screening (94.8%) at school. For AIS with a 60% risk of progression, immediate bracing (76.4%) was preferred both to observation and bracing in the event of subsequent documented worsening (21%), and to complete avoidance of bracing with a view to possible future surgery (2.6%). In cases presenting a 25% risk of progression, therapeutic exercises (86.9%) were preferred to periodic check-ups, and bracing only if scoliosis progresses (13.1%) (Table 3).

**Table 3:** Results in the full and pre-test groups: percentages (95% confidence interval) are reported. AIS: adolescent idiopathic scoliosis.

**Question**	**Answers**	**Study Sroup (SG)**	**Pre-test Group (PG)**
AIS with a 25% risk of progression	Physical exercises until growth is complete in an attempt avoid/delay bracing	86.90% (84.78–88.93)	81% (73.31–88.69)
	Periodic check-ups, bracing only if scoliosis progresses	13.10% (11.07–15.13)	19% (11.31–26.69)
AIS with a 60% risk of progression	Immediate bracing, in an attempt to avoid surgical correction	76.37% (73.17–79.56)	70% (67.25–72.75)
	Periodic check-ups, bracing only if scoliosis progresses	21.04% (17.97–24.10)	28% (25.29–30.71)
	No bracing at all, periodic check-ups, surgical correction if scoliosis progresses	2.59% (1.37–3.82)	2% (0.81–3.19)
School screening for AIS	To screen	94.8% (93.45–96.15)	96% (92.16–99.84)
	Not to screen	5.2% (3.85–6.55)	4% (0.16–7.84)
Relatives – friends with AIS	Yes	32.31% (29.47–35.15)	36% (26.56–45.51)
	No	67.69% (64.85–70.53)	64% (54.59–73.41)
Prior knowledge of treatments for AIS	Yes	43.55% (40.53–46.56)	33% (23.78–42.22)
	No	56.45% (53.44–59.47)	67% (57.58–76.22)

The responses in the SG were not statistically significantly associated with the gender of the responders; neither were they dependent upon the school or the responder's level of education. Opinions on screening were not associated with prior knowledge of AIS or AIS treatments.

## Discussion

People have preventive attitudes: they prefer conservative treatment, but they do not necessarily choose the easiest method or the least aggressive approach. Faced with a 60% probability of progression, immediate bracing was preferred to observation and bracing in the event of subsequent documented worsening. Although the lack of scientific evidence for or against physical exercises was stressed [19], as were the costs and psychological burden involved generated by physical exercises, the latter nevertheless emerged as the preferred method of preventing a worsening of AIS in which there is a 25% risk of progression. Screening was strongly favoured, even after presentation of all the limitations that have prompted many authors to consider it inefficient and, ultimately, of no use [20].

One major problem of the study was the low response rate in the SG. The low response rate was not found to be influenced by the different methods used to motivate and educate responders (headmaster's letter to parents – in the SG – *versus *a direct meeting and explanation if needed – in the PG) and is thus probably attributable to the complexity of the questionnaire. The questionnaire was perceived as "complex" by the majority of participants in the PG groups, and questions were changed according to suggestions received to reduce as much as possible this complexity. Anyway, being this study some kind of a "role playing" in which parents had to face an hypothetical situation of pathology of their children that they never thought of before, we both expected, and accepted some degree of complexity due to the need of understanding the situation, the actual literature evidence, as well as "pros and cons" of possible interventions (Table 1). Anyway one of the aim of the study was to be as much neutral as possible, avoiding any interaction between researchers and families (aside from the questionnaire), so to avoid possible influences on the answers, and the methodology was developed accordingly. Being not satisfied with the response rate of less than 40% in the SG, we introduced a direct comparison with the PG, that hadn't been planned at the start of the study and otherwise we would have avoided. This anyway allowed to verify that the data might, given the similarity of the patterns and sizes of the responses obtained in the two groups, be deemed socially (and clinically, although not statistically) relevant. Moreover, given the similarity of the two group, it could be argued also that the "external interference" applied in this case in the PG group did not have a strong influence on final results.

As stated in the method section, we avoided asking to parents of children already affected by scoliosis to avoid biased responses. The bias that can be introduced in this case is that parents of patients have already formed their ideas, that usually develops from discussions with physicians, radiologists, orthotists, physios, other patients, etc.: moreover, treatments proposed and accepted in this case will for sure drive the thoughts of parents. In this way, this study is some kind of a "role playing" in normal people, asking them to put themselves in the situation as if they had a child with scoliosis: 40% of a large sample accepted to play this game. Moreover, social acceptability relates to the entire society, and is not what is usually proposed in clinics, i.e. "patient preference"[17]. Another study could (and should) be done in the "biased" sample of scoliosis parents and children to verify their opinions and preferences, but in this case this should be matched and compared with the opinion of the treating team. We have also to point out that presenting the actual evidence, "pros and cons" of treatments to children with scoliosis and their families could represent an external interference with treatment already proposed, possibly being unethical.

Another possible weakness of the study could be not having questioned children directly and alone (they were required to answer together with their parents). We avoided that because of the complexity of the questionnaire, and the fact that usually final decisions on treatment, at the age considered (11–13 years), are reached by parents, eventually together with their children.

The risks of progression considered (25% and 60%) do not match to what have recently been proposed in the International SOSORT Guidelines for scoliosis [29] because this study have been planned and performed much before. At that time we have chosen 25% risk of progression because it means that you have a low risk, but still some: are people interested in doing some treatment instead of the "wait and see" usual approach even with this so low risk ? Moreover, 60% risk of progression means that you are at risk, more than 50%, but not in any case very high: do people even in this low-degree risk prefer bracing instead of nothing ? These were the answers we were seeking for with this study.

These results, which may seem obvious, are here presented, for the first time, in numerical terms. Acceptability studies generally involve difficult individual decisions, and are conducted in intensive care or surgical settings [17]: this study proposes a new, social approach to the question of acceptability of treatments [30]. Its main limitations are the low response rate in the SG; the "laboratory" setting (questionnaire, normal subjects), the use of proxy responders (parents);the cultural bias, which could be peculiar to northern Italy.

## Conclusion

Even though conservative options for AIS treatment may be more costly, more time-consuming, and generate a greater psychological burden on families and their physicians than more aggressive approaches [4,6], families still appear to have conservative attitudes. These results may have several implications for clinicians, researchers and policy-makers. On the clinical side, when proposing a treatment for AIS, the patient's preferences should carefully be considered, discussing the different therapeutic options [23,24] according to the actual evidence[11]; several decision aids have been developed in literature [31], and one relating to surgery for AIS can be found on the Internet [31]. On the scientific side, the current consensus [5,6,32], and increasing focus of research (Table 4), in favour of aggressive AIS treatments should be carefully reconsidered: our results reveal a gap separating the scientific and everyday worlds. Policy-makers should take into account these results when making decisions about school screening and research funding.

**Table 4:** Evolution of indexed literature on idiopathic scoliosis treatment from the period 1970–1974 to the period 1999–2004, based on a Medline search. All the indexed literature is considered, plus the contents of the journal "Spine", whose impact factors is one of the highest in this field.

			**Papers published in each period of five years**					
			
			1970–74		1980–84		1999–2003	
	
	**First term**	**AND**	N°	%	N°	%	N°	%
**Medline**	"Idiopathic scoliosis"		59		243		495	
	"Idiopathic scoliosis"	surgery	20	33,9%	65	26,7%	268	54,1%
	"Idiopathic scoliosis"	"conservative treatment" OR brace OR braces OR exercise OR exercises	19	32,2%	44	18,1%	94	19,0%
**Journal "Spine" (founded in 1978)**	"Idiopathic scoliosis"				40		198	
	"Idiopathic scoliosis"	surgery			10	25,0%	135	68,2%
	"Idiopathic scoliosis"	"conservative treatment" OR brace OR braces OR exercise OR exercises			9	22,5%	27	13,6%

Future research should verify these results in other contexts and with different, possibly less complex instruments.

## Authors' contributions

Both authors contributed to all phases of research

*Appendix 1. The original questionnaire used for the study*

See Additional file 1

*Appendix 2. English translation of the original questionnaire used for the study. This translation has not been validated in English: it is proposed only to help the readers understand better the methods used. If other researchers would like to use this questionnaire, we strongly suggest to go through a formal trans-cultural validation process.*

See Additional file 2

## Supplementary Material

Additional file 1 "Appendix 1. The original questionnaire used for the study." Click here for file

Additional file 2 "Appendix 2. English translation of the original questionnaire used for the study. This translation has not been validated in English: it is proposed only to help the readers understand better the methods used. If other researchers would like to use this questionnaire, we strongly suggest to go through a formal trans-cultural validation process." Click here for file
