# Supplementary material for: Social acceptability of treatments for adolescent idiopathic scoliosis: a cross-sectional study
Source: Scoliosis. 2006 Aug 24;1:14. doi: 10.1186/1748-7161-1-14 (PMC1560163; doi:10.1186/1748-7161-1-14)
Supplement: Additional file 1 — "Appendix 1. The original questionnaire used for the study." [file 1748-7161-1-14-S1.doc]

Egr. Sig., Gent.le Sig.ra

le chiediamo un po’ del suo tempo per aiutarci a prendere le migliori decisioni terapeutiche per i nostri giovani pazienti affetti dalla scoliosi. Infatti, come sempre in medicina, anche in questo campo esistono delle scuole di pensiero: queste si basano però sull’idea dei medici, più che sul parere dei pazienti. Vogliamo sapere da lei, che non ha una figlia/o con la scoliosi, cosa farebbe se si trovasse di fronte alle scelte che un medico di norma fa. Potremo così sapere qual è il punto di vista dei pazienti e di conseguenza avere uno strumento prezioso per orientare le nostre scelte terapeutiche. Grazie

**N.B. Se suo figlio/a ha già la scoliosi**, avendo già un medico di riferimento, la invitiamo a barrare la casella accanto ( ) e a non prendere in considerazione il resto del questionario.

Grazie

Stefano Negrini

Responsabile Sezione di Riabilitazione Ortopedica - Unità di Riabilitazione Fondazione Don Gnocchi ONLUS, IRCCS

(Istituto di Ricerca), Milano

***Cos’è la scoliosi idiopatica****: è una patologia che provoca una deformazione della colonna vertebrale che progressivamente si aggrava durante la crescita. Se la scoliosi supera un certo livello dà problemi di salute: conseguenze estetiche ed un possibile peggioramento in età adulta con dolori e deformazioni progressiva. Le terapie che vengono proposte durano sino alla fine della crescita (età tra i 16 ed i 18 anni) o cambiano se si constata un peggioramento.*

## Legga le situazioni seguenti e scelga l’opzione che preferisce

## Situazione 1

Alla visita un medico di sua fiducia riscontra una **scoliosi lieve**.

La scoliosi di sua/o figlia/o ha circa il **25% di probabilità di peggiorare** durante il periodo residuo di crescita.

- Se la scoliosi peggiora: sarà necessario usare un **corsetto sino a fine crescita**. *Il corsetto è una struttura rigida in plastica aderente al tronco (resta sotto i vestiti) che consente di continuare le attività quotidiane (sport incluso), sia pure con delle difficoltà. Si porta tutto il giorno (notte sempre inclusa) per 6-12 mesi, quindi si diminuiscono progressivamente le ore diurne.*
- Se la scoliosi non peggiora: non causerà **nessun problema di salute** né ora né in età adulta.

| **Le alternative** | **Le conoscenze scientifiche oggi** | Vantaggi | **Svantaggi** |
| --- | --- | --- | --- |
| Non fare **nulla** e ricontrollare periodicamente la situazione | Non ci sono studi che forniscano gli elementi per prevedere con certezza se una scoliosi peggiorerà. | Se la scoliosi non peggiora: non abbiamo perso tempo e denaro sottoponendo nostra/o figlia/o ad una terapia inutile e noiosa. | Se la scoliosi peggiora: non abbiamo cercato di evitare, o ritardare di qualche mese l’uso del corsetto |
| Fare **ginnastica** **sino a fine crescita** a scopo preventivo.  *Sono esercizi svolti dal ragazzo con un programma individuale, sotto la guida di un insegnante specializzato, presso appositi Centri. La terapia di norma avviene per 1 ora circa, 2 volte alla settimana, in gruppo con altri ragazzi.* | ⇒ Non ci sono studi che dimostrino con certezza che la ginnastica serve o che la ginnastica non serve nella cura della scoliosi.  ⇒ Ci sono alcuni studi che suggeriscono che la ginnastica possa essere utile a frenare l’evoluzione della scoliosi. | La ginnastica serve Se la scoliosi non peggiora: abbiamo usato una terapia che potrebbe aver frenato la scoliosi.  Se la scoliosi peggiora: abbiamo usato una terapia che potrebbe aver rallentato l’evoluzione della scoliosi ritardando di qualche mese l’uso del corsetto | **La ginnastica non serve**  Sia che la scoliosi peggiori o non peggiori: abbiamo perso tempo e denaro sottoponendo nostra/o figlia/o ad una terapia inutile e noiosa. |

### Cosa sceglierei per mia/o figlia/o

- Non farei fare a mia/o figlia/o la ginnastica a scopo preventivo, per ridurre la possibilità di dover usare il corsetto, o anche solo per ritardare di qualche mese l’uso del corsetto.
- Farei fare a mia/o figlia/o la ginnastica. Farei controllare mia/o figlia/o periodicamente dal medico di mia fiducia e, se la scoliosi peggiora, gli farei usare il corsetto.

## Situazione 2

Alla visita un medico di sua fiducia riscontra una **scoliosi di media entità**.

La scoliosi di sua/o figlia/o ha circa il **60% di probabilità di peggiorare** progressivamente durante il periodo residuo di crescita.

- Se la scoliosi peggiora: sarà necessario ricorrere ad un **intervento chirurgico**. *L’intervento è una procedura chirurgica importante, con possibili effetti collaterali che arrivano sino alla rara paralisi o al rarissimo decesso. Risolve la scoliosi usando del materiale metallico da fissare alle vertebre coinvolte, che vengono bloccate tra di loro: la colonna diventa un unico osso e i movimenti si effettuano solo tra le vertebre rimaste libere. È possibile continuare a muoversi, ma non più con la zona della colonna che è stata bloccata.*
- Se la scoliosi non peggiora: ci sono **meno probabilità che causi problemi di salute** ora ed in età adulta.

| **Le alternative** | **Le conoscenze scientifiche oggi** | **Vantaggi** | **Svantaggi** |
| --- | --- | --- | --- |
| Non fare **nulla** e ricontrollare periodicamente la situazione | Come sopra | | |
| Indossare un **corsetto sino a fine crescita.**  *Cos’è e come viene portato il corsetto è stato spiegato sopra (Situazione 1, dati iniziali)* | ⇒ Ci sono studi che dimostrano con sufficiente certezza che i corsetti servono per la cura della scoliosi  ⇒ Il corsetto è più efficace se la scoliosi è meno grave  ⇒ Il corsetto non serve a tutti i pazienti | Il corsetto è servito Se la scoliosi non peggiora: abbiamo usato una terapia che potrebbe aver bloccato la scoliosi.  Se la scoliosi peggiora: abbiamo usato una terapia che potrebbe aver ridotto il peggioramento della scoliosi | **Il corsetto non è servito**  Abbiamo perso tempo e denaro sottoponendo nostra/o figlia/o ad una terapia che si è rivelata per lui inutile, oltre ad essere psicologicamente pesante.. |

### Cosa sceglierei per mia/o figlia/o

- Farei mettere a mia/o figlia/o il corsetto per ridurre la possibilità di subire un intervento chirurgico.
- Non farei mettere a mia/o figlia/o il corsetto. Farei ricontrollare mia/o figlia/o periodicamente dal medico e, se la scoliosi peggiora, a quel punto gli farei mettere il corsetto. So che così le probabilità di ottenere un risultato con il corsetto diminuiscono, perché peggiore è la scoliosi, minori sono le probabilità di evitare l’intervento.
- Non farei mettere a mia/o figlia/o il corsetto. Farei ricontrollare mia/o figlia/o periodicamente dal medico e, se la scoliosi peggiora, gli farei l’intervento chirurgico.

## Risponda per favore a quest’ultima domanda

Fare uno screening scolastico significa effettuare la visita sistematica di tutti i ragazzi a scuola per verificare se esiste la scoliosi. Oggi in molte ASL lo screening non viene più fatto perché prevale uno dei due pareri medici contrapposti.

- I detrattori dello screening pensano che il suo costo superi i benefici; ritengono che si riscontrino troppo frequentemente scoliosi in ragazzi in cui non peggiorerà e quindi si effettuino troppi trattamenti inutili.
- I sostenitori dello screening dicono che è con lo screening è possibile individuare la scoliosi in molti pazienti quando non è grave e, con il trattamento, ridurre la possibilità di peggioramento e quindi evitare terapie più pesanti.

(corsetto, intervento chirurgico).

### La mia scelta

- Secondo me lo screening non deve essere fatto. E’ inutile continuare a spendere denaro per la prevenzione se non ci sono prove assolute che i soldi vengano spesi bene. Meglio risparmiare a diversi ragazzi di sapere inutilmente di avere la scoliosi e sopportare che alcuni di loro debbano subire trattamenti pesanti (corsetto, intervento).
- Secondo me lo screening deve essere fatto comunque. Meglio investire denaro per prevenire che spendere per curare. Non importa se diversi ragazzi sapranno inutilmente di avere la scoliosi, se possiamo evitare ad alcuni di loro trattamenti più pesanti.

## Alcuni vostri dati generali

|  | Età | Sesso | Titolo di studio |  |  | Età | Sesso | Classe frequentata |
| --- | --- | --- | --- | --- | --- | --- | --- | --- |
| Compilatore del questionario |  |  |  |  | Figlio 1 |  |  |  |
|  |  |  |  |  | Figlio2 |  |  |  |
| Coniuge/convivente |  |  |  |  | Figlio3 |  |  |  |

Qualche familiare, parente amico/a a lei vicino soffre o ha sofferto di scoliosi? Sì No

Prima di questo questionario, aveva già un’idea sul trattamento secondo lei più giusto per la scoliosi? Sì No
