# Supplementary material for: Social acceptability of treatments for adolescent idiopathic scoliosis: a cross-sectional study
Source: Scoliosis. 2006 Aug 24;1:14. doi: 10.1186/1748-7161-1-14 (PMC1560163; doi:10.1186/1748-7161-1-14)
Supplement: Additional file 2 — "Appendix 2. English translation of the original questionnaire used for the study. This translation has not been validated in English: it is proposed only to help the readers understand better the methods used. If other researchers would like to use this questionnaire, we strongly suggest to go through a formal trans-cultural validation process." [file 1748-7161-1-14-S2.doc]

Dear Sir / Madam,

we are requesting a little bit of your time to assist us in taking the best therapeutic decisions for our young scoliotic patients. Indeed, as always happens in medicine, in this field too there are many schools of thought that are based more on the physicians’ ideas, than on patients’ opinions. Since you don’t have a son / daughter suffering from scoliosis, we would like to know what you would do in case you had to face the choices a physician normally has to make. In this way we could know what is the patients’ viewpoint and consequently have a valuable tool to direct our therapeutic choices. Many thanks.

**N.B. If your son / daughter already has scoliosis**, having a doctor in charge, we ask you to tick the nearby box ( ⬜ ) and not to consider the rest of the questionnaire.

Thanks.

Stefano Negrini

Responsible of the research project

***What idiopathic scoliosis is****: it is a pathology that determines a deformation of the vertebral column, that progressively worsens with growth. If scoliosis exceeds a certain level, it creates health problems, cosmetic consequences and a possible worsening in adult age with pain and progressive deformation. Proposed therapies last until the end of growth (age 16 - 18 years) or change if a worsening is seen.*

## Please, read the following situations and choose the option you prefer

## Situation # 1

At examination, your physician finds a **light scoliosis**.

Your son’s / daugther’s scoliosis has roughly a **25% likelihood of worsening** during the remaining part of growth.

- If scoliosis worsens: it will be necessary to use a **brace until the end of growth**. *The brace is a rigid plastic structure which is adherent to the whole trunk (and is hidden under dresses); it allows to keep on doing the usual daily activities (including sport), even if with difficulties. It must be worn all day long (including the night) for 6-12 months. Thereafter, daytime wearing hours are progressively diminished.*
- If scoliosis doesn’t worsen: it will **not cause health problems**, neither now nor in adult age.

| **Alternatives** | **Today’s scientific knowledge** | Advantages | **Disadvantages** |
| --- | --- | --- | --- |
| Doing **nothing** and periodically checking the situation. | There are no studies providing elements in order to predict with certainty if a scoliosis will worsen. | If scoliosis doesn’t worsen: we did not lose time and money subjecting our son / daughter to a useless and tiresome therapy. | If scoliosis worsens: we did not try to avoid or delay of some months the recourse to brace. |
| Doing **exercises until the end of growth**, as a preventive measure.  *These are exercises done by the child basing on an individual schedule, under the guidance of a specialized teacher, at suitable Centers. The therapy session normally lasts 1 hour and is done 2 times a week in a group with other children.* | ⇒ There are no studies proving with certainty that exercises are useful or useless for scoliosis treatment.  ⇒ There are some studies suggesting that exercises can be useful to slow down scoliosis progression. | Exercises are useful If scoliosis doesn’t worsen: we used a therapy that could have slowed down scoliosis progression.  If scoliosis worsens: we used a therapy that could have slowed down scoliosis progression by delaying of some months the recourse to brace. | **Exercises are useless**  Whether scoliosis worsens or not: we lost time and money subjecting our son / daughter to a useless and tiresome therapy. |

### What I would choose for my son / daugther

- I would not get my son / daughter to do exercises as a preventive measure, with the idea of reducing the probability of using the brace or even only to delay of some months the recourse to brace.
- I would get my son / daughter to do exercises. I would have him / her periodically examined by my physician and, if scoliosis progresses, I would get him / her to wear the brace.

## Situation # 2

At examination, your physician finds a **medium magnitude scoliosis**.

Your son’s / daugther’s scoliosis has roughly a **60% likelihood of worsening** during the remaining part of growth.

- If scoliosis worsens: a **surgical intervention** will be necessary. *This operation is a major surgical procedure, with possible side effects that very rarely arrive to paralysis and almost never to death. It requires the use of metal material that will be fixed to vertebrae involved in the scoliosis; these vertebrae are blocked with each other: the spine becomes a unique bone and movements are possible only for the vertebrae that were left free. It is still possible to move, but no more with the spinal segments that have been blocked.*
- If scoliosis doesn’t worsen: **it will be less likely to cause health problems**, neither now nor in adult age.

| **Alternatives** | **Today’s scientific knowledge** | **Advantages** | **Disadvantages** |
| --- | --- | --- | --- |
| Doing **nothing** and periodically checking the situation. | Same as above. | | |
| Wearing a **brace until the end of growth.**  *What is a brace and how it is worn has been explained above (Situation # 1, initial data)* | ⇒ Some studies prove with enough certainty that braces are useful for scoliosis treatment.  ⇒ Brace is more effective if scoliosis is not very severe.  ⇒ Brace is not useful for all patients. | Brace has been useful If scoliosis doesn’t worsen: we used a therapy that could have blocked scoliosis progression.  If scoliosis worsens: we used a therapy that could have reduced scoliosis progression. | **Brace has been useless**  We lost time and money subjecting our son / daughter to a therapy that turned out to be useless, besides being psychologically heavy. |

### What I would choose for my son / daugther

- I would get my son / daughter to wear the brace, with the idea of reducing the likelihood of undergoing surgery.
- I would not get my son / daughter to wear the brace. I would have him / her periodically examined by my physician and, if scoliosis progresses, at that point I would get him / her to wear the brace. I know that in this case the likelihood of obtaining results with bracing diminishes, because the worse the scoliosis, the smaller the likelihood of avoiding surgery.
- I would not get my son / daughter to wear the brace. I would have him / her periodically examined by my physician and, if scoliosis progresses, I would get him / her to undergo surgery.

## Please answer this last question

Doing a school screening means doing a systematic examination of all schoolchildren in order to verify the presence of scoliosis. Today, at many Local Health Authorities, screening is no more done, because 1 of 2 medical views prevails.

- Screening detractors think that its cost exceeds its benefits; they maintain that scoliosis is too frequently seen in children who will not worsen and so a high number of useless treatments could be avoided.
- Screening supporters maintain that through screening it is possible to detect scoliosis in many patients when it is not severe and, with treatment, to reduce the likelihood of worsening and so to avoid heavier therapies (brace, surgery).

### My choice

- In my opinion, screening should not be done. It has no sense to go on spending money for prevention if we do not have definite proofs that money is well spent. It is better to save many children the trouble of uselessly knowing of having scoliosis and of being sometimes subjected to heavy treatments (brace, surgery).
- In my opinion, screening is to be done anyway. It is better to invest money to prevent, than spend it to cure. It doesn’t matter if some children will uselessly know of having scoliosis, if we can avoid some of them heavier treatments.

## Some general details about you

|  | Age | Gender | Educ. qual. |  |  | Age | Gender | Attended grade |
| --- | --- | --- | --- | --- | --- | --- | --- | --- |
| Questionnaire compiler |  |  |  |  | Child # 1 |  |  |  |
|  |  |  |  |  | Child # 2 |  |  |  |
| Spouse / cohabitant |  |  |  |  | Child # 3 |  |  |  |

A family member, relative, friend has or has had scoliosis? Yes ⬜ No ⬜

Before receiving this questionnaire, did you already have an idea on

the treatment you considered the best one for scoliosis? Yes ⬜ No ⬜
